# Influence of Plant Additives on Changes in the Composition of Fatty Acids, Lipid Quality Indices and Minerals of Fermented Dairy Products from Cow’s Milk

**DOI:** 10.3390/molecules30020235

**Published:** 2025-01-09

**Authors:** Beata Paszczyk, Elżbieta Tońska

**Affiliations:** Department of Commodity and Food Analysis, The Faculty of Food Sciences, University of Warmia and Mazury in Olsztyn, 10-726 Olsztyn, Poland; elzbieta.tonska@uwm.edu.pl

**Keywords:** yogurts, plant additives, fatty acids, lipid quality indices, minerals

## Abstract

The aim of this study was to assess the effect of selected plant additives on changes in the content of fatty acids, lipid quality indicators and mineral composition of yogurts produced from cow’s milk. The analysis included natural yogurts and yogurts enriched with 10% of chia seeds, hulled hemp seeds, quinoa seeds and oat bran. The fatty acid composition, the content of lipid quality indicators and the content of mineral components was varied in all analyzed yogurts. The plant additives used caused significant (*p* ≤ 0.05) changes in their fatty acid content, i.e., a significant decrease in the content of saturated fatty acids (SFAs) and short-chain fatty acids (SCFAs), and a significant increase in the content of monounsaturated fatty acids (MUFAs) and polyunsaturated fatty acids (PUFAs). The plant additives used caused significant (*p* ≤ 0.05) changes in the content of fatty acids, i.e., a significant decrease in the content of saturated fatty acids (SFAs) and short-chain fatty acids (SCFAs), and a significant increase in the content of monounsaturated fatty acids (MUFAs) and polyunsaturated fatty acids (PUFAs). It was shown that additives such as chia seeds and peeled hemp seeds caused the greatest changes in the analyzed yogurts. Yogurts with these additives were characterized by a significant increase in the content of polyunsaturated fatty acids (PUFAs), including *n*-3 acids, and a more favorable *n*-6/*n*-3 ratio. Yogurts with these additives were also characterized by significantly (*p* ≤ 0.05) lower atherogenic (AI) and thrombogenic (TI) indices and a higher hypocholesterolemia-to-hypercholesterolemia ratio (H/H). The addition of peeled hemp seeds caused the greatest changes in the content of minerals. Yogurts with hemp seeds were characterized by the highest content of all measured macroelements, as well as copper, iron and zinc. In turn, the highest manganese content was determined in the yogurts with the addition of chia seeds.

## 1. Introduction

Milk and dairy products play an important role in the healthy daily diet of the general population. They are rich in many nutrients, including various minerals, vitamins, fatty acids and high-quality protein. The content of all nutrients in dairy products depends mainly on their content in the milk they are made of, the production process applied and the additives used. Milk fat is one of the most complex natural fats, consisting of approximately 400–500 fatty acids [1,2]. Bovine milk contains approximately 60–70% of saturated fatty acids (SFAs), ca. 20–35% monounsaturated fatty acids (MUFAs), of which the most important is oleic acid, and approximately 3% polyunsaturated fatty acids (PUFAs), including *n*-3 and *n*-6 fatty acids in the optimal physiological ratio [3,4,5]. The composition of fatty acids plays an important role in shaping the taste and consistency of yogurts, which largely determines their nutritional value. According to research sources [6,7], fatty acids present in the milk fat of ruminants can have a positive or negative effect on consumer health [8,9]. For example, short-chain fatty acids (SCFAs), including butyric acid (C4:0), are considered a modulator of the gene function that plays an important role in cancer prevention, eliciting a positive effect on human health. In turn, C8:0 and C10:0 fatty acids are known for their antiviral effects [10]. Oleic acid (*cis*9 C18:1), the major MUFA in milk fat, enhances the activity of low-density lipoprotein receptors and reduces serum cholesterol concentration [11]. *Trans* 11 C18:1 acid, the major *trans* C18:1 isomer in milk fat, exerts anticarcinogenic and antiatherosclerotic effects [12]. Conjugated linoleic acid *cis*9*trans*11 C18:2 (CLA) present in milk fat has numerous health-promoting properties [13,14,15,16]. Furthermore, *n*-3 polyunsaturated fatty acids may be helpful in preventing heart disease and improve the immune response. Linolenic acid (C18:3) exhibits anticancer and antiatherosclerotic properties [17,18]. In turn, (*n*-6) PUFAs have been shown to improve insulin sensitivity and thus reduce the incidence of type 2 diabetes [19].

Yogurt is a nutrient-rich product produced by bacterial fermentation of milk. It provides many nutrients that can help improve the health of compromised groups. It is rich in calcium, vitamin D, riboflavin, vitamin B6 and vitamin B12. It supports intestinal microbiota as a carrier of probiotics, which control intestinal infections, lower the blood cholesterol level and lactose intolerance, and reduce the risk of development of many diseases. The consumption of yogurt reduces the risk of type 2 diabetes, improves insulin resistance, and lowers the blood levels of glucose and triglycerides [20,21]. Yogurts can be divided into two groups: the first group includes standard yogurts produced by fermentation, whereas the second group is represented by bio- or probiotic yogurts. Standard yogurt is produced upon the activity of *Lactobacillus bulgaricus* and *Streptococcus thermophilus*. These bacteria suppress the putrefactive-type fermentations of the intestinal flora, which helps maintain the overall health of the digestive tract, while bio-yogurts refer to those that are made with probiotic strains of *Bifidobacteria* and *Lactobacillus acidophilus*, which have numerous health benefits [22,23]. In addition to this classification, yogurts available on the market come in a wide range of flavors, textures and forms to meet a variety of consumer preferences. They can be consumed as a snack, dessert or part of a meal, and can also be used to prepare various dishes, sauces or cakes. Many different ingredients are added to yogurts, including popular fruits, fruit seed extracts, vegetables, nuts, muesli and spices, in order to attract consumer attention, reduce the sour taste and enhance the health-promoting effect [24,25]. Hlédika and Lógó [26] have reported that the most popular products among consumers are those containing fruit juices or purees from strawberries, forest fruits, peaches, cherries and raspberries. Yogurts containing rare fruit species, such as pomegranate and jacaranda seeds, mulberry fruits or goji berries, are less available [27,28]. The additives used not only improve sensory and functional properties of yogurts by modifying their taste, color and texture, but also provide many bioactive components [25]. Chia seeds, shelled hemp seeds and quinoa are characterized by a high nutritional value and health properties. One of the main reasons why these seeds are becoming increasingly popular is the lipid content and the quality of this lipid fraction related to the composition of fatty acids. Over 90% of the fatty acids present in hemp seed oil are unsaturated fatty acids with a unique *n*-6/*n*-3 ratio [29]. Chia seeds are characterized by a high content of polyunsaturated fatty acids, mainly α-linolenic acid (ALA), which constitutes about 60% of all fatty acids, have a high content of *n*-3 acids and a favorable ratio of *n*-6 to *n*-3 acids, owing to which they have a beneficial effect on improving the blood lipid profile [30]. Chia seeds provide many minerals and vitamins, mainly vitamin B1, B2 and niacin. Quinoa is rich in unsaturated fatty acids such as oleic (C18:1), linoleic (C18:2) and linolenic (C18:3). The share of these fatty acids in quinoa constitutes 87–88% of the total fatty acids in the seed. [31]. Vega-Gálvez et al. report that the mineral content in quinoa seeds is higher than that in other cereals [32]. Oats and oat products, including oat bran, are an excellent source of valuable compounds that can be successfully used in various industrial sectors, as well as in the prevention and treatment of many diseases [33].

In Europe, consumer demand for healthier food products is successively growing, with a focus on nutritional aspects. Increased health awareness has led to an increase in the consumption of functional dairy products, and more specifically, yogurts enriched with health-promoting nutrients.

The aim of the present study was, therefore, to assess the effect of selected plant additives on changes in the content of fatty acids, lipid quality indicators and mineral composition of yogurts produced from cow’s milk.

## 2. Results and Discussion

### 2.1. Fatty Acid Composition

Fat extracted from the analyzed yogurts was characterized by diversified contents of fatty acids (Table 1). Saturated fatty acids (SFAs) prevailed in all analyzed products. The highest SFA content was determined in the fat from natural yogurts (63.16% of total fatty acids). In turn, the fat extracted from the analyzed yogurts with additives had a significantly lower (*p* ≤ 0.05) content of these acids. The content of monounsaturated fatty acids (MUFAs) was the highest in the fat from the yogurts with oat bran (24.75% of total fatty acids), while the lowest was determined in the fat extracted from yogurts with added hulled hemp seeds (19.46% of total fatty acids). The highest content of PUFAs (27.97% of total fatty acids) was determined in the fat from yogurts with hulled hemp seeds. Significantly lower (*p* ≤ 0.05) contents of PUFAs were found in the other analyzed yogurts. The lowest content of these acids was found in natural yogurts (2.51% of total fatty acids). The present study’s results indicate that the fat extracted from yogurts with added chia seeds had a significantly higher (*p* ≤ 0.05) content of *n*-3 (13.81% of total fatty acids) and that fat from the yogurts with shelled hemp seeds had a significantly higher (*p* ≤ 0.05) content of *n*-6 PUFAs (19.51% of total fatty acids) compared to the other yogurts tested (Table 1). The lowest content of *n*-3 and *n*-6 was determined in the fat from natural yogurts (0.33% and 1.28% of total fatty acids, respectively). Some cereal additives used, among others, in the dairy industry have high nutritional potential; for example, chia seeds contain 25–35% fat, the vast majority of which is polyunsaturated acids. Chia is a plant with the highest known percentage of α-linolenic acid, which reaches as much as 68% of total fatty acids [34]. A higher PUFA content, mainly α-linolenic acid, in yogurts with the addition of chia seeds and strawberries compared to natural yogurts was shown by Kowaleski et al. [35]. The highest content of this acids was detected in yogurts with 14% chia and 8% strawberries added. Derewiaka et al. [36] also obtained an increase in the content of polyunsaturated fatty acids (PUFAs) in their study with yogurt enriched with chia seed oil. Hemp seeds are also rich in healthy lipids with a high content of polyunsaturated fatty acids, such as linoleic acid (*n*-6), α-linolenic acid (*n*-3) and some vitamins (vitamins E, D and A) [37]. In turn, quinoa grains are rich in unsaturated fats, mainly linolenic, oleic and linoleic acids, with polyunsaturated fatty acids accounting for 87–88% of their total fatty acids. Quinoa grain consumption helps lower blood cholesterol and reduces the risk of development of atherosclerosis and other circulatory system diseases [38]. *n*-3 and *n*-6 fatty acids cannot be synthesized in the body and need to be supplied to the body with the diet. As demonstrated in a variety of studies [39,40,41,42,43,44,45], an adequate intake of these acids is associated with a reduced risk of many diseases. *n*-6 and *n*-3 acids act antagonistically to each other, i.e., *n*-6 acids exert pro-inflammatory and prothrombotic effects, while *n*-3 acids elicit anti-inflammatory effects and inhibit platelet aggregation [46]. In addition, a properly balanced diet should provide an appropriate ratio of these acids. Too many *n*-6 fatty acids and a high *n*-6/*n*-3 ratio promote the pathogenesis of many diseases. An increased level of *n*-3 fatty acids (lower *n*-6/*n*-3 ratio) has the opposite effect [43,47]. In the analyzed fermented products, the *n*-6/*n*-3 ratio was the lowest in the fat extracted from yogurts with chia seeds (0.387), whereas the highest (8.44) was observed in the fat from yogurts with oat bran (Table 1). In the fat from yogurts analyzed by Paszczyk and Łuczyńska [48], it was 4.77 in natural yogurts, 3.04 in bio-yogurts and up to 10.59 in fat from yogurts with fruit and cereal grains. 

A lower *n*-6/*n*-3 ratio in dairy products indicates a better composition of fatty acids and confirms that these products can better support proper body functions. The average European’s diet is poor in *n*-3 acids. Enriching yogurts with chia seeds or peeled hemp seeds, which are a valuable source of these acids, will, on the one hand, increase the fat content of these products, but on the other hand, significantly increase the content of *n*-3 acids, which will have a beneficial effect on our health. It is therefore worth drawing attention to these seeds and promoting their use in the daily diet. A diet based on a regular supply of *n*-3 fatty acids, dietary fiber, protein, vitamins, minerals and antioxidants plays a key role in preventing many diseases of the modern world.

### 2.2. The Lipid Quality Indices

The results regarding lipid quality indices—DFA, OFA, AI, TI and H/H—for the individual analyzed yogurts are summarized in Table 2. The desired fatty acid (DFA) index is the sum of MUFAs, PUFAs and stearic acid (C18:0). The DFA values differed between natural yoghurts and yoghurts with additives. In natural yoghurts, the DFA index was, on average, 34.95. In the yoghurts with additives included in this study, the DFA values were significantly higher (*p* ≤ 0.05). The highest DFA levels were recorded in yoghurts with the addition of peeled hemp seeds (53.86). Higher DFA index values in natural yoghurts compared to enriched yogurts were noted in earlier studies by Paszczyk and Czarnowska-Kujawska [49]. Hypercholesterolemic fatty acids (OFAs) are the sum of SFAs—C18:0. In the conducted studies, the OFA index was the highest in natural yogurts, amounting to 54.63. In enriched yogurts, the OFA values were significantly lower (*p* ≤ 0.05), ranging from 38.89 in yogurts with the addition of hulled hemp seeds to 53.58 in yogurts with the addition of quinoa (Table 2). The average AI value for natural yogurts was 3.46, and for enriched yogurts, it was significantly lower (*p* ≤ 0.05), ranging from 1.30 in yogurts with hulled hemp seeds to 3.05 for yogurts with quinoa. Higher AI values in natural yogurts compared to enriched yogurts were obtained by Paszczyk and Czarnowska-Kujawska [49]. The lowest value of the thrombogenicity index (TI) (0.81) was determined in the fat from yogurts with chia seeds (Table 2), whereas the fat from all other analyzed yogurts showed significantly higher values (*p* ≤ 0.05). The AI and TI take into account the different effects of individual fatty acids on the likelihood of developing cardiovascular diseases, such as atherosclerosis and coronary artery disease [8]. The AI value indicates the relationship between the sum of the main SFAs and the sum of the main MUFAs. The TI, in turn, indicates the ratio of procoagulant fatty acids (SFAs) to anticoagulant fatty acids (MUFAs, *n*-3 and *n*-6 PUFAs). Higher values of these indicators indicate a higher risk of developing cardiovascular diseases [50]. In turn, the H/H index describes the relationship between hypocholesterolemic fatty acids (C18:1 and PUFAs) and hypercholesterolemic fatty acids (C12:0, C14:0 and C16:0). In natural yogurts, the H/H index was the lowest and amounted to 0.37, and in enriched yogurts, it was significantly higher (*p* ≤ 0.05), ranging from 0.43 in yogurts with quinoa to the highest value, 1.17, in yogurts with the addition of hulled hemp seeds. In the fat from yogurts with additives (muesli, cereal grains), analyzed by Paszczyk and Czarnowska-Kujawska [49], the H/H was 0.70, whereas it was significantly lower in other analyzed yogurts, ranging from 0.41 in bio-yogurts to 0.47 in eco yogurts.

### 2.3. Mineral Composition

The elements found in the largest amounts in the tested samples were calcium, potassium and phosphorus. The average contents of these elements in natural yogurts were 309.7 mg/100 g of product, 185.7 mg/100 g of product and 131.6 mg/100 g of product, respectively (Table 3). Our previous study [51] showed a lower calcium content (188.71 mg/100 g) and a higher potassium content (209.49 mg/100 g) and phosphorus content (143.11 mg/100 g) in yogurts obtained from cow’s milk in laboratory conditions. The content of these elements in cow’s milk yogurts was also analyzed by Mohammed et al. [52], who determined them in cow’s yogurts at 120.33 mg/100 g (calcium), 136.87 mg/100 g (potassium) and 85.33 mg/100 g (phosphorus). Differences in the mineral content in yogurts may result from the composition and quality of the milk used for production, the composition of the yogurt starter, and the method of yogurt production. These factors may affect the higher or lower content of minerals in the finished product [53,54]. The study conducted showed a significantly lower (*p* ≤ 0.05) calcium content in the yogurts with added chia seeds, quinoa and oat bran. In the yogurts with added shelled hemp seeds, the calcium content was at a level similar to that determined in natural yogurts. The results given in Table 4 indicate that chia seeds, oat bran, and quinoa contain large amounts of fiber, which may limit the availability of calcium and other minerals, such as iron, zinc, and copper. A significantly higher (*p* ≤ 0.05) potassium content was found in all enriched yogurts than in natural yogurts. A significantly (*p* ≤ 0.05) increased phosphorus content was found only in the yogurts with the addition of hulled hemp seeds (Table 3). This study showed that 10 g of peeled hemp seeds added to 100 g of yogurt increased the content of this element by over 61 mg in 100 g of the product. Phosphorus is an important nutrient in our diet. It plays a key role as an important physiological buffer and, together with calcium, as a component of bone minerals in the skeleton. Phosphorus is a common element of the diet. It is a natural component of food, but it is also used in the food industry as an additive with stabilizing and thickening properties. The body’s demand for this element depends on many factors (age, gender, and physiological condition) [55]. When establishing a diet, it should be taken into account that both high and low phosphorus contents in the diet can cause adverse health effects. To protect yourself from exceeding the daily dose of phosphorus supplied with food, you should limit, among others, highly processed products in your diet. The lowest contents of minerals in the natural yogurts tested were found for manganese (0.002 mg/100 g) and copper (0.009 mg/100 g). A similar copper content (0.012 mg/100 g) in cow’s milk yogurts was shown in our previous study (Paszczyk et al. [51]). The content of these elements varied in the enriched yogurts (Table 3). All enriched yogurts had significantly higher (*p* ≤ 0.05) manganese and copper contents. The highest manganese content was found in the yogurts with added chia seeds (0.102 mg/100 g), and the highest copper content in those with added hulled hemp seeds (0.06 mg/100 g). The sodium (Na) content was the highest in the yogurts with the addition of hulled hemp (54.98 mg/100 g), while in the remaining enriched yogurts and natural yogurts, its content remained at a similar level. The yogurts with the addition of hulled hemp seeds were also characterized by the highest content of zinc (0.914 mg/100 g), iron (0.226 mg/100 g) and magnesium (36.08 mg/100 g). In turn, the lowest iron content (0.077 mg/100 g) was found in natural yogurts, that of magnesium (15.88 mg/100 g) in the yogurts with added oat bran, and that of zinc (0.562 mg/100 g) in the yogurts with added quinoa. The observed changes in the mineral content of fortified yogurts compared to natural yogurts may be due to the different amounts of these minerals in these seeds. The mineral content present in seeds may vary significantly depending on environmental conditions, soil mineral composition, applied fertilizers and plant variety. Farinon et al. [56] reported that the mineral content of peeled hemp seeds ranged from 890 to 1170 mg per 100 g of seeds for P, K: 250–2821, Mg: 237–694, Ca: 90–955, Na: 6.8–27, Fe: 4–240, Mn: 4–15, Zn: 4–11, Cu: 0.5–2 and Cd: 0.0015–0.4. In turn, Lan et al. [57] indicate a high variability in the content of mineral components in peeled hemp depending on the plant variety and year of harvest.

Chia seeds were already consumed thousands of years ago. Current research results indicate their high nutritive value and confirm their extensive health-promoting properties. Chia seeds contain many minerals. Kulczyński et al. [30] have reported that chia seeds are super-rich in potassium, magnesium, calcium and phosphorus, with their contents reaching 407–726 mg/100 g, 335–449 mg/100 g, 456–631 mg/100 g and 860–919 mg/100 g, respectively. In addition, chia seeds have 1.8, 6.0 and 2.4 times more iron than lentils, spinach and liver [58,59]. Studies on the effect of chia seed addition on the quality of yogurts were conducted by, among others, Mouss et al. [60], Kowaleski et al. [35] and Nakov et al. [61]. The authors state that the quality of yogurts with added chia seeds depends mainly on the concentration of these seeds in the product and the storage time. Moussa et al. [60] assessed the effect of adding chia seeds in the amount of 1.5%, 3%, 4.5% and 6% on the biochemical, biofunctional, microbiological and sensory parameters of yogurt during 28-day storage under refrigerated conditions. The studies of these authors showed that the best parameters were characterized by yogurts containing 1.5 or 3% of chia seeds. In turn, Kowaleski et al. [35] conducted a study aimed at developing recipes for probiotic yogurts with the addition of strawberries and chia seeds in different proportions and assessing the effect of different concentrations of these products on the content of lipids, protein, dietary fiber, pH, acidity, apparent viscosity, fatty acids, mineral profile, lactic acid bacteria and bifidobacteria during 35 days of yogurt storage, as well as assessing the acceptability of these yogurts by consumers through sensory analysis. The prepared yogurt samples contained appropriate proportions of chia seeds and strawberries: control sample (0% chia, 26% strawberry), 1 (6% chia, 8% strawberry), 2 (14% chia, 8% strawberry), 3 (6% chia, 12% strawberry), 4 (14% chia, 12% strawberry), and 5 (10% chia, 10% strawberry). The authors’ studies showed that the addition of chia increased the levels of protein, lipids, dietary fiber and polyunsaturated fatty acids (PUFAs), especially omega-3 acids, and had an impact on the mineral content. The sensory acceptability of the products was proportional to the addition of strawberries and inversely proportional to the addition of chia. The formula containing 6% chia and 12% strawberries achieved an acceptability score of >70% and was considered the best yogurt formula due to its high nutritional value. Nakov et al. [61] showed that adding chia seeds to yogurt at a concentration of 5% significantly affected both the nutritional and sensory properties of the product during storage. Sensory evaluation showed that yogurts with 5% chia seeds had better sensory properties than those with higher concentrations. Yogurts with 10% chia seeds showed a significant deterioration of sensory properties during storage. Ayaz et al. [62] assessed the effect of chia seed consumption as a mid-morning snack on short-term satiety and mood. Their study showed that the consumption of 7 g or 14 g of chia seeds with plain yogurt as a mid-morning snack increased satiety. Furthermore, chia seed consumption had no effect on mood. Chia seeds are classified as a novel food and according to the Commission Implementing Regulation (EU) 2017/2470, the daily intake of chia seeds should not exceed 15 g. Also, hemp seeds have recently spurred increasing interest as a valuable ingredient for producing high-quality food and dietary supplements due to their exceptional nutritional value. They are also a good source of macro- and microelements (4–7.6%), including P, K, Mg, Ca and Na, as well as Fe, Mn, Zn and Cu. The contents of these elements present in hemp seeds varies significantly depending on various parameters, such as environmental conditions, soil mineral composition, fertilizers and plant variety [63,64,65]. Quinoa, in turn, is a very good source of manganese and copper. Calcium from quinoa is better absorbed than calcium from milk, which is why it is recommended for people struggling with lactose intolerance [66,67]. The evaluation of the effect of quinoa flour addition on the nutritional properties of yogurts was conducted by Rafiq et al. [68]. The prepared yogurts with quinoa flour added in the amount of 0.5%, 1.0%, 1.5% and 2.0% were stored in a refrigerator for 28 days and then their properties were evaluated. The microbiological and physicochemical properties of all evaluated variants were encouraging. However, the sensory profile showed that 1% quinoa flour addition was the best.

## 3. Materials and Methods

### 3.1. Samples

The research material consisted of yogurts produced using a starter culture of the company “VIVO” containing strains of probiotic bacteria: *Bifidobacterium lactis* (2 strains), *Bifidobacterium infantis*, *Lactobacillus bulgaricus*, *Lactobacillus acidophilus* (2 strains), *Streptococcus thermophilus*, *Lactobacillus paracasei*. The yogurts used for the analyses were produced from commercial milk using the thermostatic method on a semi-technical scale at the Department of Dairy and Quality Management, UWM in Olsztyn. The production process included the following steps: the milk was heated to 45 °C, and then centrifuged and degassed (80 kPa; 60 °C). The milk was pasteurized using the HTST method (72 °C/15 s; ALFA-LAVAL P20-HB pasteurizer, Lund, Sweden) and allowed to cool to 6 °C (ALFA-LAVAL P20-HB pasteurizer, Lund, Sweden). After the cooling process, skimmed milk was added to the milk to standardize the fat content to 2 ± 0.1%. Then, the material was homogenized in a two-stage process (18/5 MPa, 65 °C; CN003 homogenizer, Spomasz Bełżyce, Bełżyce, Poland) and then pasteurized in long-term VHT (Very High Temperature) pasteurization (90 °C/5 min; ALFA-LAVAL P20-HB pasteurizer, Lund, Sweden). The milk prepared in this way was cooled to 45 °C and inoculated. Then, the yogurts were packed into individual containers and left to mature in thermostats (Binder GF115, Tuttingen, Germany) for about 4 h at 43.5 °C until they reached pH 4.6. Then, chia seeds, hulled hemp seeds, quinoa seeds and oat bran were added to the produced yogurt in an amount of 10%. Freshly prepared natural and enriched yogurts were analyzed. All the plant additives used were purchased from Bio Planet (Leszno, Poland), a company which offers organic food. All their products have organic certificates. The analysis included natural yogurts and enriched yogurts. Table 4 shows the nutritional value of plant additives used in accordance with the declaration provided by the manufacturer on the packaging.

### 3.2. Fatty Acid Analysis

#### 3.2.1. Fat Extraction

Fat was extracted from the analyzed yogurts with the method of Folch [69]. The procedure was performed in accordance with the method described by Paszczyk and Czarnowska-Kujawska [47].

#### 3.2.2. Preparation of Fatty Acid Methyl Esters

The fatty acid methyl esters (FAMEs) were prepared according to the International Dairy Federation method (IDF 182:2002) [70]. n-Hexane and 2 M KOH in methanol were added to the fat sample, and the mixture was shaken for 1 min and then left for 5 more minutes. Then, sodium hydrogen sulfate (NaHSO_4_) was added, and the mixture was centrifuged for 3 min (1000 spins/min). The top layer of the prepared methyl esters was taken for chromatographic analysis.

#### 3.2.3. Gas Chromatography (GC) Analysis

Chromatographic separation of the obtained methyl esters was carried out by gas chromatography (GC) using a Hewlett-Packard 6890 GC System (Műnster, Germany) chromatograph with a flame ionization detector (FID) and a capillary column (Chrompack, Middelburg, The Netherlands) with CP Sil 88 phase (length 100 m, internal diameter 0.25 mm, film thickness 0.2 μm). The separation conditions are described in Table 5.

The identification of fatty acids (FAs) was carried out using analytical standards and data from the literature [71,72,73]. Reference milk fat (BCR Reference Materials) with the symbol CRM 164 (Aldrich, Taufkirchen, Germany) and methyl ester standards (Sigma-Aldrich, Steinheim, Germany and Supelco, Bellefonte, PA, USA) were used. The proportions of individual FAs were calculated based on the ratio of their peak areas to the total area of all identified acids (% mass fraction).

#### 3.2.4. The Lipid Quality Indices

Lipid quality indices were calculated based on equations posited by Medeiros et al. [74] and Paszczyk and Tońska [75]:

DFA = UFA + C18:0

Hypercholesterolemic fatty acids (OFAs):

OFA = C12:0 + C14:0 + C16:0

The values of the atherogenicity index (AI) and thrombogenicity index (TI) were calculated using the formulas proposed by Ulbricht and Southgate [8] and Osmari et al. [76]:

AI = (C12:0 + (4 × C14:0) + C16:0)/(*n*-3 PUFA + *n*-6 PUFA + MUFA)

TI = (C14:0 + C16:0 + C18:0)/((0.5 × C18:1) + (0.5 × sum of other MUFA) + (0.5 × *n*-6 PUFA) + (3 × *n*-3 PUFA) + (*n*-3 PUFA/*n*-6 PUFA))

The hypocholesterolemic/hypercholesterolemic ratio (H/H) was calculated according to the work of Ivanova and Hadzhinikolova [50]:

H/H = (C18:1*n*-9 + C18:2*n*-6 + C18:3*n*-3)/(C12:0 + C14:0 + C16:0)

### 3.3. Analysis of Minerals

Yogurt samples were wet-washed in a mixture of nitric and perchloric acid (3:1; *v*/*v* Suprapure, Merck, Darmstadt, Germany) on an aluminum heating block (DK 20, VELP Scientifica, Usmate, Italy) for 4–5 h, gradually increasing the temperature from 120 to 200 °C. The colorless digest was placed in 25 mL volumetric flasks, to which deionized water was added for determination. Individual minerals (magnesium, manganese, zinc, copper, iron, and calcium) were determined by flame atomic absorption spectrometry (air-acetylene flame) using an iCE 3000 Series atomic absorption spectrometer (Thermo-Scientific, Waltham, MA, USA) with a Glite data station, background correction (deuterium lamp), and appropriate cathode-ray tubes. Selected elements were determined at the following wavelengths (nm): Mg—285.2, Mn—279.5, Zn—213.9, Cu—324.8, Fe—248.3 and Ca—422.7. Potassium and sodium were analyzed by atomic emission spectrometry (AES), while the P content was determined by the colorimetric method (P—610 nm) with ammonium molybdate, sodium sulfate and hydroquinone. Absorbance measurements were performed using VIS 6000 spectrophotometer (Thermo Scientific, Madison, WI, USA).

### 3.4. Statistical Analysis

The statistical analyses were conducted using STATISTICA ver. 13.1 software (Statsoft, Kraków, Poland) [77]. The one-way analysis of variance ANOVA (Duncan’s test) was used to test significant differences. Differences were found significant at *p* ≤ 0.05.

## 4. Conclusions

Yogurts are very popular among consumers around the world. These contain a number of nutrients important in the human diet. Food fortification is one of the most important processes, improving the quality and quantity of its nutrients. Such fortification can have a significant impact on consumer health. Our study shows that adding chia seeds, hemp seeds, quinoa and oat bran to yogurt affects the fatty acid content, lipid quality index values and mineral content. The addition of chia increased the levels of polyunsaturated fatty acids (PUFAs), especially *n*-3 fatty acids and minerals (potassium, phosphorus, copper, iron and manganese). Yogurts with added chia had the lowest *n*-6/*n*-3 ratio and the lowest index of thrombogenicity (TI). Yogurts with added shelled hemp had a higher content of PUFAs, including the highest content of *n*-6 and all determined macroelements, and the highest content of copper, iron and zinc. These yogurts were characterized by the highest content of hypocholesterolemic fatty acids (DFAs) and the lowest content of hypercholesterolemic fatty acids (OFAs). In turn, the addition of quinoa and oat bran caused a slight increase in the content of PUFAs, including *n*-6 acids. These yogurts were characterized by a higher content of copper, manganese, potassium, and a lower content of phosphorus and calcium compared to natural yogurts. These results may be important for consumers when choosing products to plan a rational diet rich in appropriate nutrients. They may also be very important for manufacturers who, in order to meet consumer demands, are constantly looking for innovative products, including those that have a more beneficial effect on health. Some cereal products, including chia seeds, quinoa or peeled hemp seeds, can be a valuable addition to yogurt because they offer functional benefits and increase the nutritional value of the final product. However, the concentration of these seeds must be carefully tested to ensure that the final product will have high sensory quality and optimal health benefits throughout its shelf life.

## Figures and Tables

**Table 1:** Fatty acid composition (% of total fatty acids) in fat extracted from the analyzed yogurts.

Fatty Acids		Natural Yogurts	Yogurts with Added Chia Seeds	Yogurts with Added Hulled Hemp Seeds	Yogurts with Added Quinoa	Yogurts with Oat Bran
ΣSCFAs	Mean	10.37 ^a^	7.69 ^d^	6.61 ^e^	8.88 ^c^	9.69 ^b^
	SD	0.15	0.14	016	0.36	0.22
ΣSFAs	Mean	63.16 ^a^	52.23 ^d^	45.31 ^e^	61.90 ^b^	60.94 ^c^
	SD	0.05	0.19	0.22	0.27	0.20
ΣMUFAs	Mean	23.91 ^b^	20.35 ^c^	19.46 ^d^	24.37 ^a,b^	24.75 ^a^
	SD	0.19	0.29	0.04	0.10	0.32
ΣPUFAs	Mean	2.51 ^e^	19.73 ^b^	27.97 ^a^	4.84 ^c^	4.54 ^d^
	SD	0.02	0.09	0.05	0.09	0.05
*n*-3	Mean	0.33 ^e^	13.81 ^a^	7.79 ^b^	0.58 ^c^	0.39 ^d^
	SD	0.01	0.02	0.01	0.02	0.01
*n*-6	Mean	1.28 ^d^	5.13 ^b^	19.51 ^a^	3.32 ^c^	3.29 ^c^
	SD	0.01	0.06	0.08	0.01	0.04
*n*-6/*n*-3	Mean	3.88 ^c^	0.37 ^e^	2.50 ^d^	5.73 ^b^	8.44 ^a^
	SD	0.11	0.01	0.01	0.16	0.40

Values are expressed as means (*n* = 3) ± standard deviation; ^a,b,c,d,e^—values in rows with different letters differ significantly (*p* ≤ 0.05); ΣSCFAs—sum of short-chain fatty acids (C4:0–C10:0); ΣSFAs—sum of medium- and long-chain saturated fatty acids; ΣMUFAs—sum of monounsaturated fatty acids; ΣPUFAs—sum of polyunsaturated fatty acids.

**Table 2:** Nutritional indices determined in the analyzed yogurts.

Fatty Acids		Natural Yogurts	Yogurts with Added Chia Seeds	Yogurts with Added Hulled Hemp Seeds	Yogurts with Added Quinoa	Yogurts with Oat Bran
DFAs	Mean	34.95 ^d^	47.60 ^b^	53.86 ^a^	37.54 ^c^	37.33 ^c^
	SD	0.27	0.34	0.12	0.17	0.49
OFAs	Mean	54.63 ^a^	44.71 ^d^	38.89 ^e^	53.58 ^b^	52.91 ^c^
	SD	0.13	0.21	0.22	0.25	0.32
AI	Mean	3.46 ^a^	1.81 ^c^	1.30 ^d^	3.05 ^b^	3.00 ^b^
	SD	0.06	0.03	0.01	0.01	0.07
TI	Mean	3.66 ^a^	0.81 ^d^	0.91 ^c^	3.42 ^b^	3.46 ^b^
	SD	0.04	0.01	0.01	0.02	0.03
H/H	Mean	0.37 ^d^	0.81 ^b^	1.17 ^a^	0.43 ^c^	0.44 ^c^
	SD	0.01	0.01	0.01	0.01	0.01

Values are expressed as means (*n* = 3) ± standard deviation; ^a,b,c,d,e^—values in rows with different letters differ significantly (*p* ≤ 0.05); DFAs—hypocholesterolemic fatty acids (ΣUFAs + C18:0); OFAs—hypercholesterolemic fatty acids (ΣSFAs-C18:0); AI (Index of Atherogenicity); TI (Index of Thrombogenicity); H/H (hypocholesterolemic/hypercholesterolemic ratio).

**Table 3:** Mineral content in the analyzed yogurts (mg/100 g of product) (Mean ± SD).

Fatty Acids		Natural Yogurts	Yogurts with Added Chia Seeds	Yogurts with Added Shelled Hemp Seeds	Yogurts with Added Quinoa	Yogurts with Oat Bran
Cu	Mean	0.009 ^e^	0.041 ^b^	0.060 ^a^	0.021 ^c^	0.018 ^d^
	SD	0.001	0.001	0.002	0.001	0.001
Mn	Mean	0.002 ^d^	0.102 ^a^	0.094 ^b^	0.053 ^c^	0.056 ^c^
	SD	0.0002	0.004	0.003	0.0004	0.001
Fe	Mean	0.077 ^c^	0.115 ^b^	0.226 ^a^	0.117 ^b^	0.086 ^c^
	SD	0.006	0.008	0.009	0.011	0.002
Zn	Mean	0.647 ^b^	0.607 ^c^	0.914 ^a^	0.562 ^d^	0.567 ^d^
	SD	0.057	0.002	0.009	0.002	0.010
Mg	Mean	25.33 ^b^	19.70 ^c^	36.08 ^a^	16.37 ^d^	15.88 ^d^
	SD	1.417	0.33	1.08	0.63	0.11
Ca	Mean	309.7 ^a^	274.5 ^b^	325.7 ^a^	256.5 ^b^	249.6 ^b^
	SD	43.87	3.6	9.0	5.9	2.1
Na	Mean	47.39 ^b^	47.07 ^b^	54.98 ^a^	47.41 ^b^	45.95 ^b^
	SD	2.298	0.88	0.49	0.39	1.25
K	Mean	185.7 ^c^	205.2 ^b^	222.2 ^a^	201.5 ^b^	200.3 ^b^
	SD	2.862	6.3	11.5	8.7	4.4
P	Mean	131.6 ^bc^	138.93 ^b^	193.08 ^a^	126.86 ^c^	126.24 ^c^
	SD	2.192	2.16	12.42	3.81	0.23

Values are expressed as means (*n* = 3) ± standard deviation; ^a,b,c,d,e^—values in rows with different letters differ significantly (*p* ≤ 0.05).

**Table 4:** Nutritional value of the plant additives used.

	Chia Seeds	Shelled Hemp Seeds	Quinoa	Oat Bran
Energy value	1824 kJ/ 443 kcal	2455 kJ/ 592 kcal	1546 kJ/ 367 kcal	1570 kJ/ 374.03 kcal
Fat (%)	31.0	48.0	6.5	9.81
Carbohydrates (%)	2.0	3.1	60.0	51.73
Fiber (%)	38.0	6.0	7.2	14.53
Protein (%)	20.0	34.0	14.0	12.44
Salt (%)	0.05	0.02	0.09	0.09

**Table 5:** Parameters of chromatographic analysis of fatty acids.

Parameter	Value
temperature column	60 °C (1 min)–180 °C, Δt = 5 °C/min
temperature detector	250 °C
temperature injection	225 °C
helium flow	1.5 mL/min
sample injection	0.4 μL, split: 50:1

## Data Availability

Data are contained within the article.
